# HIV-1 Vif global diversity and possible APOBEC-mediated response since 1980

**DOI:** 10.1093/ve/veae108

**Published:** 2024-12-12

**Authors:** Eric Lewitus, Yifan Li, Morgane Rolland

**Affiliations:** U.S. Military HIV Research Program, Walter Reed Army Institute of Research, 503 Robert Grant Ave, Silver Spring, MD 20910, USA; Henry M. Jackson Foundation for the Advancement of Military Medicine, Inc., 6720A Rockledge Dr, Bethesda, MD 20817, USA; U.S. Military HIV Research Program, Walter Reed Army Institute of Research, 503 Robert Grant Ave, Silver Spring, MD 20910, USA; Henry M. Jackson Foundation for the Advancement of Military Medicine, Inc., 6720A Rockledge Dr, Bethesda, MD 20817, USA; U.S. Military HIV Research Program, Walter Reed Army Institute of Research, 503 Robert Grant Ave, Silver Spring, MD 20910, USA; Henry M. Jackson Foundation for the Advancement of Military Medicine, Inc., 6720A Rockledge Dr, Bethesda, MD 20817, USA

**Keywords:** HIV-1, Vif, hypermutation, phylogenetics, APOBEC3

## Abstract

HIV-1 Vif’s principal function is to counter the antiretroviral activities of DNA-editing APOBEC3 cytidine deaminases. Unconstrained APOBEC3 activity introduces premature stop codons in HIV-1 genes and can lead to viral inactivation. To investigate the evolution and diversification of Vif over the HIV-1 pandemic and document evidence of APOBEC3-mediated pressure, we analyzed 4612 publicly available sequences derived from 10 dominant subtypes and circulating recombinant forms (CRFs) using the Hervé platform. We found widespread evidence of diversifying selection that was convergent across subtypes and CRFs, but remarkable stability in consensus sequences over time. Divergence and selection did not favor APOBEC3-interacting sites. We furthermore found that APOBEC3-induced substitutions in *env* and *gag-pol* genes increased over time and were positively associated with *vif* diversity. These results suggest that APOBEC3-driven adaptation in Vif is relatively rare and that permissiveness to human APOBEC3-induced substitution as a mechanism for generating diversity may be advantageous to HIV-1 evolution.

## Introduction

The HIV-1 Vif protein is a small accessory protein that is unique to lentiviruses (Malmquist et al. 1969). While the molecular interactions of Vif differ across lentiviral species, particularly between those infecting primates and other mammals (Kane et al. 2015, Yoshikawa et al. 2016), the function of the protein to counteract host antiviral factors is conserved (Sheehy et al. 2002, Zheng et al. 2017). Apolipoprotein B mRNA-editing catalytic polypeptide (APOBEC) cytidine deaminases belong to a family of DNA/RNA-editing enzymes, the most abundant of which are human APOBEC3A (hA3) proteins arrayed on human chromosome 22 (Jarmuz et al. 2002), that are typically incorporated into nascent virions and cause extensive C-to-U deamination (Zhang et al. 2003). These APOBEC-guided plus-strand G-to-A mutations target 5ʹ GG or GA bases, which often, but not inevitably, result in premature stop codons and potential viral inactivation (Yu et al. 2004). A sequence with extensive APOBEC-induced mutations, defined by the proportion of GRD-to-ARD compared to GY-to-AY (or GRC-to-ARC), that lead to potential viral inactivation is termed a hypermutant. HIV-1 Vif targets APOBEC3 deaminases for proteasomal degradation to prevent virion encapsidation (Sheehy et al. 2003); however, Vif counteraction of APOBEC3 enzymes is inefficient and hypermutants are observed in people living with HIV-1 (PLWH) (Janini et al. 2001, Simon et al. 2005).

Early adaptation of Vif to APOBEC3 homologs in other primate species prior to human zoonosis may have primed HIV-1 Vif to effectively counteract human hA3 and left little necessity, or opportunity, for further adaptation (Richards et al. 2015). Multiple transmissions of simian immunodeficiency virus (SIV) from nonhuman apes to humans (Sharp and Hahn 2011), as well as similar Vif-APOBEC3G binding affinities between HIV-1 and SIV (Letko et al. 2013), imply that Vif was already adapted to at least partially neutralize hA3 activity (Nakano et al. 2020). However, naturally occurring amino acid (AA) mutations at many sites are associated with differentiated hA3 inactivation (Stephens et al. 2001, He et al. 2008, Mulder et al. 2008, Ooms et al. 2013); and subtype-specific N-terminal domain variability in Vif is a key determinant of hA3F and hA3G binding activity (Chen et al. 2009, Dang et al. 2009, Iwabu et al. 2010).

Mutations in Vif that result in sporadic inactivation of its anti-hA3 efficiency have been implicated in intensifying HIV-1 diversity through nonlethal hypermutation (Simon et al. 2005). This complicates how beneficial or deleterious any Vif mutation may be. For example, mutations identified in motifs consistent with hA3-mediated hypermutation have been linked to drug resistance (Berkhout and Ronde 2004, Haché et al. 2006, Mulder et al. 2008, Pillai et al. 2008) and immune escape (Wood et al. 2009). While the inheritance of defective Vif alleles has no demonstrated effect *in vivo* on disease progression (Piantadosi et al. 2009, Kikuchi et al. 2015), it remains unknown how competition with hA3 in PLWH has shaped Vif diversification population-wide.

In this work, we investigated the diversification of Vif in seven subtypes and three circulating recombinant forms (CRFs) and the prevalence of hA3-induced substitution in *env* and *gag-pol* between 1980 and 2022. We hypothesized that competition between Vif and hA3 enzymes would favor selection at hA3-mediated sites in Vif and have a negative effect on the number of hA3-induced substitutions occurring over time in *env* and *gag-pol*. Over 4000 publicly available Vif and 3000 *env* and *gag-pol* sequences coupled with the Hervé platform (Lewitus et al. 2024), a dynamic web interface for exploring HIV-1 sequence diversity, allowed us to elucidate the temporal dynamics of Vif evolution and its effect on HIV-1 diversification.

## Materials and methods

### Data collection and eligibility criteria

Vif AA alignments were retrieved from the Los Alamos National Laboratory (LANL) HIV-1 sequence database on 5 December 2023 for subtypes A1, A6, B, C, D, F1, and G and CRFs 01_AE, 02_AG, and 07_BC (https://www.hiv.lanl.gov/components/sequence/HIV/search/search.html). One sequence per individual was selected. Sequences were removed if they had no sampling year or country information or an incomplete open reading frame. When two sequences shared ≥ 98% identity, the later-sampled sequence was removed. To confirm that a ≥ 98% deduplication threshold was sufficient to remove linked sequences (e.g., sequences sampled years apart from the same individual), we calculated the distribution of pairwise sequence identity and counted the number of sequence pairs in each subtype/CRF with ≥ 98% sequence similarity (Supplementary Fig. S1). We also calculated the median pairwise diversity for sequences in each subtype/CRF corresponding to sequence identity thresholds between 92% and 100% (Supplementary Fig. S2). Hypermutants were determined by hypermutR (Labuschagne et al. 2017, Rose and Korber 2000) using a one-sided Fisher’s exact test with *P* <0 .1.

The Vif 2004 and 2021 consensus sequences for each subtype/CRF were downloaded from LANL. Nucleotide sequences with accession IDs corresponding to the curated Vif AA sequences were retrieved from LANL for *vif*, *gag-pol*, and *env*.

For *vif*, *gag-pol*, and *env*, the pairwise distance between all sequences was calculated with the R package phangorn (Schliep et al. 2017). If the median distance of a sequence to sequences of its assigned subtype/CRF was larger than that to sequences of another subtype/CRF, then that sequence was considered to have been mis-subtyped and removed. Similarly, we analyzed each subtype/CRF alignment for outlying sequences. We first calculated the minimum branch length between each phylogenetic tip and its closest neighbor and then performed a Rosner’s test (Rosner 1975) on the distribution. We then computed the pairwise distance between each nucleotide sequence and its subtype/CRF consensus to determine if potential outliers were >3 standard deviations from the median.

### Phylogenetic reconstruction

Subtype/CRF alignments were aligned to each other one at a time, beginning with subtype B, to construct an alignment of all Vif subtypes/CRFs. Alignments were done using MAFFT v7.475 (Katoh and Standley 2013) and recombination breakpoints were identified using GARD (Kosakovsky Pond et al. 2006), which searches for phylogenetic incongruence among partitions of the alignment. For each protein, a phylogeny was constructed with IQ-TREE 2 (Minh et al. 2020) based on the best-fit model as determined by the Bayesian information criterion (BIC) and data partitioned according to the inferred recombination breakpoints.

### Consensus and ancestral sequence generation

For each subtype/CRF, we generated consensuses for sequences sampled between 1980–90, 1991–2000, 2001–10, and 2011–22 using the Hervé platform (https://hiv1.shinyapps.io/Herve) (Lewitus et al. 2024). Each consensus was determined by the most common residue at each site and ties were broken in the order of LGIKEAVTRQSPNDWYFHCM (https://www.hiv.lanl.gov/content/sequence/CONSENSUS/AdvCon.html). Sites with > 50% gaps in the subtype/CRF alignment were excluded. Ten sequences were required for generating a consensus. Ancestral sequences were reconstructed at all internal nodes using FastML v.3.11 with branch-length optimization and a gamma distribution (Ashkenazy et al. 2012) for each subtype/CRF alignment using sequences sampled between 1980 and 2000 when >50 sequences were available in that sampling period and between 1980 and 2022 otherwise. Differences between circulating sequences and the sequence of the most recent common ancestor (MRCA) were determined using the AA reconstructions with the highest marginal probability at each AA site.

### Diversity and divergence estimates within subtypes and CRFs

For each subtype/CRF, the divergence between the MRCA and each sequence was calculated with a HIV-1 between-patient matrix (Nickle et al. 2007) and empirical base frequencies using the Hervé platform (Lewitus et al. 2024). Univariate distributions for normal, gamma, and exponential models were fit to each dataset using maximum likelihood estimation; best-fit models were determined by likelihood ratio tests. Nonlinear least-squares parameter estimation was used to compute the growth rate of AA divergence estimates as a function of sampling time and the carrying capacity inferred by the best-fit model, which here was defined as the projected maximum median divergence estimate.

Intra-subtype/CRF diversity was estimated using the pairwise distance between all sequences within a subtype/CRF and all sequences within a subtype/CRF and the subtype/CRF consensus. Pairwise diversity was calculated with a HIV-1 between-patient matrix (Nickle et al. 2007) in the R package phangorn (Schliep et al. 2017). Estimates of MRCA divergence, pairwise diversity, and growth models were additionally computed on down-sampled alignments that randomly sampled 25 sequences per sampling period for subtypes B and C and CRF01_AE.

The percentage of residues in subtype/CRF alignments matching the consensus was counted at all sites, excluding gaps. Pairwise diversity and consensus residues were computed on sequences sampled from the same sampling period.

To calculate the number of polymorphic sites in cytotoxic T lymphocyte (CTL) epitopes, CTL epitopes were predicted on each Vif subtype/CRF consensus sequence for the most frequent alleles (>5% frequency) in five human subpopulations: African/African-American, Asian/Pacific Islands, European/European descent, Middle East/North coast of Africa, and South or Central America/Hispanic/Latine (Hurley et al. 2020). Peptides with a predicted binding score ranked in the top 99.5% compared to a set of random natural peptides and with a nanomolar affinity <50 nM were considered potential CTL epitopes (i.e., strong binders). Rank scores and affinity were predicted with netMHCpan4.1 (Reynisson et al. 2020). Within each subtype/CRF, we counted the number of polymorphic sites between consensus sequences for sequential sampling periods that fell within at least one CTL epitope in the consensus sequence for the more recent sampling period. The percentage of each consensus sequence covered by predicted CTL epitopes was calculated by counting the number of unique epitopes that occurred within each consensus sequence multiplied by nine (as each epitope was a 9-mer).

### Diversifying selection analyses

Subtype/CRF *vif* nucleotide sequences were codon-aligned and stop codons were removed. Phylogenetic trees were constructed for each alignment using IQ-TREE 2 (Minh et al. 2020) with the best-fit model determined by ModelFinder (Kalyaanamoorthy et al. 2017). Sites under pervasive diversifying selection were identified using a Bayesian inference of per-site dN/dS on each alignment and corresponding phylogeny using the FUBAR tool in HyPhy (Murrell et al. 2013). Sites were considered under significant selection if the posterior probability of negative selection (dS > dN) was <0.05 and the posterior probability of positive selection (dN > dS) was >0.9. For each site under selection, the frequency distribution of residues for sequences sampled pre-2000 and post-2000 were compared using a Levene’s test to determine whether there was more variance in the distribution for sequences sampled before (*P* > 0.95) or after (*P* < 0.05) 2000; and a one-tailed Kolmogorov–Smirnov test was used to determine whether the most frequent residue had changed between sequences sampled post-2000 (*P* < 0.05).

### Hypermutation and hA3-induced substitution analyses for *env* and *gag-pol*

hA3-induced substitutions were identified in *env* and *gag-pol* alignments for each subtype/CRF using the R package hypermutR (Labuschagne et al., Rose and Korber 2000). Sequences were categorized as hypermutants with a Fisher’s exact test *P* < 0.1. hA3-induced substitutions were identified by hypermutR (typ = ‘hyp’ and muted = ‘TRUE’) and the number of sites per sequence was averaged over all sequences within a subtype/CRF. For each subtype/CRF, odds ratios and confidence intervals for an hA3-induced substitution occurring at a site in a sampling period given the number of sequences with an hA3-induced substitution at that site in a previous sampling period were derived from fitting a binomial generalized linear model. Area under the receiver operating characteristics curve (AUC) was calculated using the R package PRROC (Grau et al. 2015) for the percentage of sequences with an hA3-induced substitution at a site during a sampling period as a function of the percentage of sequences with an hA3-induced substitution at the same site in the previous sampling period. Generalized linear models and AUCs were computed for hA3-induced substitution sites in the 1991–2000 alignment as a function of the number of sequences mutated at that site in 1980–90, in 2001–10 as a function of 1991–2000, in 2011–22 as a function of 2001–10.

### *Vif* nucleotide diversity and *env* and *gag-pol* hA3-induced substitutions

Mean *vif* MRCA divergence and mean *vif* pairwise diversity was calculated for each subtype/CRF within each sampling period and regressed against the mean number of hA3-induced substitutions per *env* and *gag-pol* sequence from the same sampling period. For subtypes A1, B, and C and CRF01_AE, mean pairwise *vif* diversity and the mean number of hA3-induced substitutions per *env* and *gag-pol* sequence within 2-year sampling periods were calculated. Linear regressions were run for the mean number of hA3-induced substitutions per *env* and *gag-pol* sequence as a function of mean pairwise *vif* diversity over time.

### hA3-interacting sites database

Evidence for Vif sites that interact with hA3 was assessed in the literature based on a structural interaction between a Vif AA and hA3 derived from cryo-electron microscopy complexes (Ito et al. 2023, Kouno et al. 2023, Li et al. 2023) and/or an experimental assay showing an effect on hA3 functioning when a Vif AA was mutated (Supplementary Table S2).

## Results

### Vif sequences sampled since 1980

**Figure 1.**
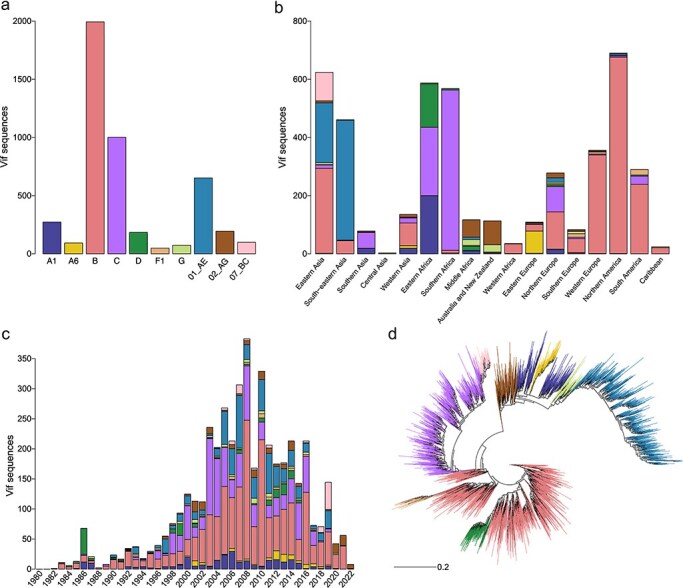
Vif sequences across subtypes/CRFs, geography, and time. The number of AA sequences sampled by (a) subtype/CRF, (b) geographical region, and (c) year. (d) Reconstructed phylogeny for Vif colored by subtype/CRF.

Our data collection yielded 10,183 Vif AA sequences derived from DNA and RNA and sampled between 1980 and 2022. After restricting to dominant subtypes and CRFs and screening sequences to ensure that only one sequence per participant was included and that each sequence had a functional open reading frame, 4612 sequences remained (Supplementary Fig. S3). These sequences corresponded to subtypes B (43.2% of sequences), C (21.7%), CRF01_AE (14.1%), A1 (5.9%), CRF02_AG (4.2%), D (4.0%), CRF07_BC (2.1%), A6 (2.0%), G (1.6%), and F1 (1.1%; Fig. 1a). Compared with the distribution of viruses found in PLWH, this represented a bias toward subtype B and against subtype C sequences (Hemelaar et al. 2019). Most sequences were sampled in Northern America (15.2%), Eastern Asia (13.7%), Eastern Africa (12.9%), and Southern Africa (12.5%; Fig. 1b). While most subtypes/CRFs were geographically isolated, subtype C was represented in Eastern and Southern Africa, Southern Asia, and Northern Europe; and subtype B was represented across Europe, Northern America, the Caribbean, and Eastern and Western Asia. Most sequences (87.8%) were sampled after 2000 (Fig. 1c) and derived from RNA (64.6%). Vif sequences were best fit by a HIVb + R10 AA substitution model (∆BIC = 402.35). Subtype and CRF sequences were generally segregated into monophyletic clades, although subtypes F1 and D were nested in subtype B, CRF07_BC was nested in subtype C, and subtype A6 was nested in A1 (Fig. 1d).

Hypermutation is deemed significant when the proportion of GRD to ARD is significantly greater than the proportion of GY to AY and GRC to ARC, as determined by a Fisher’s exact test (Harris 2008), where R = [A|G], D = [A|G|T], and Y = [C|T] following IUPAC convention. Therefore, sequences that contain hA3-induced substitutions are not necessarily deemed hypermutants.

### Divergence from the MRCA in subtype/CRF epidemics

**Figure 2.**
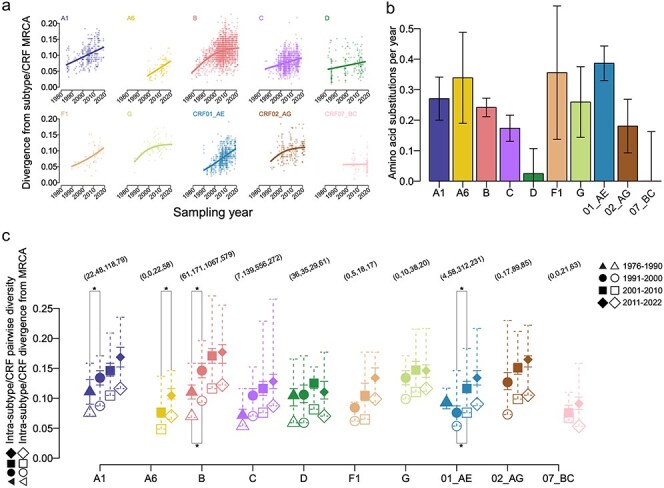
Vif MRCA divergence and pairwise diversity over time. For each subtype/CRF, (a) fitted growth curves to MRCA divergence, (b) mean AA substitutions per year (whiskers indicate 95% confidence intervals), and (c) median MRCA divergence (open shapes) and pairwise diversity (closed shapes) by decade. Solid whiskers indicate 25% and 75% quantiles and dashed whiskers indicate minimum and maximum values of pairwise diversity. Asterisks indicate significant pairwise differences between MRCA divergence (below) and pairwise diversity (above). The number of sequences in each sampling period is shown parenthetically above.

We computed the MRCA for each subtype/CRF based on sequences sampled prior to 2000 when at least 50 sequences were available and prior to 2022 otherwise. We then fitted growth models to the divergence of sequences from the MRCA as a function of sampling year for each subtype/CRF: all were best fit by a gamma model except subtype D and CRF07_BC, which were best fit by normal models (Fig. 2a). The growth rates ranged from 0 to 0.132, which corresponded to between 0.002 and 0.414 AA substitutions per year (Fig. 2b). We next calculated the model-corrected percentage of divergence from the MRCA and pairwise diversity between sequences. The median increase in MRCA divergence between 1980–90 and 1991–2000 was 0.014 (min = −0.045, max = 0.029), between 1991–2000 and 2001–10 was 0.018 (0.008, 0.028), and between 2001–10 and 2011–22 was 0.010 (−0.011, 0.020); and for median pairwise diversity was 0.033 (−0.017, 0.036), 0.024 (0.012, 0.040), and 0.012 (−0.018, 0.040) (Fig. 2c, Supplementary Table S1). There was a significant increase (Mann-Whitney U test, *P* < .05) in divergence from the MRCA between the two earliest available sampling periods for subtype B and CRF01_AE and in pairwise diversity for subtypes A1, A6, and B and CRF01_AE (Fig. 2c). This suggests that diversity increases were typically highest at the beginning of a subtype/CRF outbreak. When only RNA-derived sequences were analyzed, results were consistent for all subtypes/CRFs except subtype D (Supplementary Fig. S4), which had a higher AA substitution rate in RNA-only (median = 0.189) than in RNA + DNA (0.125); however, this difference is most likely contributable to sparsely sampled RNA-only sequences between 1990 and 2010. There was little effect on diversity estimates when the subtypes B and C and CRF01_AE alignments were down-sampled (*n* = 79–100 Vif sequences per subtype/CRF), which showed a mean difference with the full-sampled alignments in MRCA divergence of −0.7% to 0.2%, pairwise diversity of 0.1% to 1.3%, and AA substitutions per year of −0.03 to 0.12; the inferred growth models were identical.

### Polymorphisms were not disproportionately at APOBEC-interacting sites or in CTL epitopes

**Figure 3.**
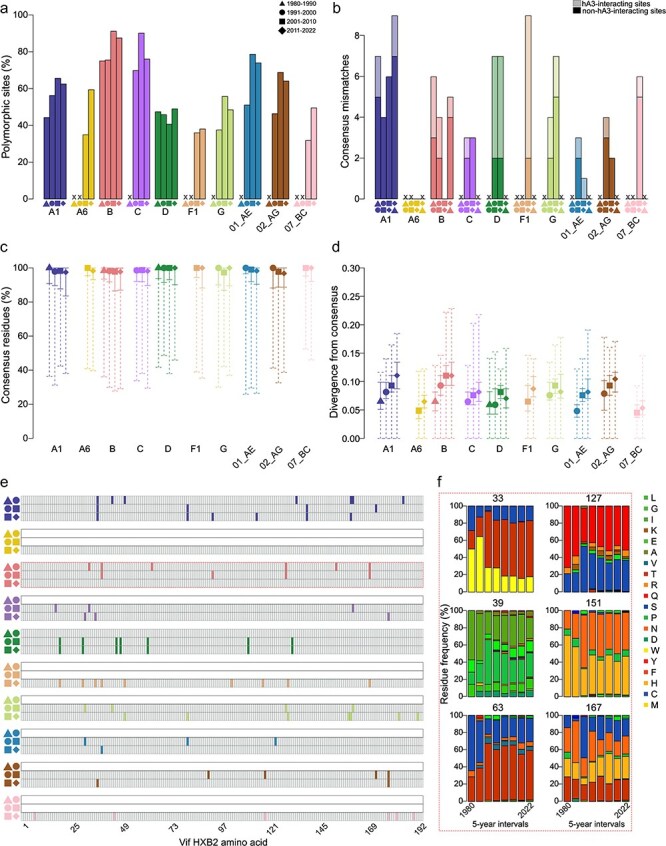
Vif polymorphisms and consensus changes over time. (a) The percentage of polymorphic sites in subtype/CRF alignments for 1980–90 (triangle), 1991–2000 (circle), 2001–10 (square), and 2011–22 (diamond). (b) The number of mismatched sites between consensuses for different sampling periods; shapes below each bar indicate the sampling periods being compared. Lighter shades represent mutations at hA3-interacting sites. Sampling periods with fewer than 10 sequences are marked with an X in (a) and (b). (c) The median percentage of consensus residues across sites for subtypes/CRFs in each sampling period. (d) Median divergence from the consensus for each sampling period across subtypes/CRFs. Solid whiskers indicate 25% and 75% quantiles and dashed whiskers indicate minimum and maximum values in (c and d). (e) Cell plots of mismatched sites between consensuses for 1980–90 and 1991–2000 (triangle-circle), 1991–2000 and 2001–10 (circle-square), and 2001–10 and 2011–22 (square-diamond) for each subtype/CRF: colored cells indicate a mismatch; gray cells indicate no mismatch; and empty cells are shown when fewer than 10 sequences were available for a subtype/CRF. Cells correspond to Vif HXB2 AA sites. (f) Stacked barplot of residue frequencies in 5-year time-windows for sites mismatched in subtype B Vif between 1980–90 and 1991–2000 (see red dashed box in panel E); the Vif HXB2 site is shown above.

We computed consensus sequences for each subtype/CRF for Vif sequences sampled between 1980–90, 1991–2000, 2001–10, and 2011–22. Consensuses were only computed when at least 10 sequences were available. Across sampling periods, 34.6%–91.2% (mean = 58.9%) of sites were polymorphic within any subtype/CRF, with an average increase of 3.7% between the first two sampling periods, 15.1% between the second and third, and 0.8% between the third and fourth (Fig. 3a). However, when we compared consensus sequences computed for each decade there were few mismatched sites over time: across subtypes/CRFs, 0–9 (mean = 2.43) sites were mismatched between sequential sampling periods (Fig. 3b). We identified in the literature 94 hA3-interacting sites (49% of Vif sites), including 18 interacting with hA3A, 46 with hA3C, 1 with hA3E, 63 with hA3F, 11 with hA3G, and 46 with hA3H (many interacted with more than one hA3 enzyme, Supplementary Table S2). Across sampling times, an average of 11.8%–77.8% of mismatches in consensus sequences per subtype/CRF occurred at hA3-interacting sites (Fig. 3b). Consensus changes were more likely to occur at hA3-interacting sites in subtypes D and F1, equally likely to occur at hA3- and non-hA3-interacting sites in subtype B, and less likely to occur at hA3-interacting sites in subtypes A1, C, and G and CRFs 01_AE, 02_AG, and 07_BC. These results were consistent when only RNA-derived sequences were included in analyses: 0–11 (mean = 5) sites were mismatched between sequential sampling periods (Supplementary Fig. S5) and included all mismatched sites in the analysis of DNA + RNA sequences.

We then predicted CTL epitopes for HLA alleles with > 5% frequency in five human subpopulations and counted the number of sites mismatched between subtype/CRF sampling period consensuses that fell within the predicted CTL epitopes. Across sampling periods, 0–17 (mean = 8.65) CTL epitopes were predicted in the subtype/CRF consensus sequences (Supplementary Fig. S6a). The average percentage of sites in CTL epitopes mismatched between consensus sequences from sequential sampling periods ranged from 0% to 100% (mean = 18.2%) (Supplementary Fig. S6b), with an average decrease over time of 2.7%. The proportion of sites mismatched between consensus sequences that were within CTL epitopes was on average 35.1% greater than expected based on the percentage of residues in consensus sequences that fell within predicted CTL epitopes (mean = 4.1%–8.1% across sampling periods, Supplementary Fig. S6c); however, this was not significantly different from 0% for any subtypes/CRF or sampling period (one-sided Mann Whitney U test, *P* > 0.52).

Across the consensus sequences for each sampling period, the consensus residue at each site represented a median of 96.8%–100% of all residues for each subtype/CRF (Fig. 3c). Therefore, while many sites were polymorphic (Fig. 3a), polymorphisms were typically restricted to only a few sequences. At sites mismatched between consensuses, however, the frequency of the consensus residue was on average 73.4% (min = 32.2%, max = 100%) overall and 71.8% (42.9%, 100%) at hA3-interacting sites. Divergence from the consensus was generally low, but increased slightly from an average of 0.057 (min = 0.043, max = 0.065) to 0.085 (0.053, 0.116) over sampling periods (Fig. 3d). Sites mismatched between consensuses across sampling periods were distributed across the Vif protein in each subtype/CRF, with no mismatched sites between 1980–90 and 1991–2000 shared between subtypes/CRFs, five (31, 80, 127, 159, 170) between 1991–2000 and 2001–10, and eleven (19, 30, 36, 37, 39, 50, 80, 128, 158, 176, 181) between 2001–10 and 2011–22 (Fig. 3e). We then traced the representation of residues at mismatched sites across consensus sequences over time. The dominant residue in any time-window was represented at a frequency of 32.2%–100% (mean of median = 81.3%) for all sites and 73.6% (42.8%, 100%) for hA3-interacting sites. At one-third of sites mismatched between consensuses, the dominant residue occurred at <60% frequency, disclosing little inclination toward a particular residue (Fig. 3f, Supplementary Fig. S7).

When we compared our consensuses to those generated by the Los Alamos National Laboratory (LANL) HIV database for 2004 and 2021 (Linchangco et al. 2022), there was an average of 1.5–5.1 and 1.3–4.5 differences, respectively, across sampling periods (Supplementary Fig. S8a and b). The LANL2004 and LANL2021 mismatches were typically at variable sites with ≥ 45.9% and ≥ 51.2% nonconsensus residues in population alignments, respectively (Supplementary Fig. S8c and d).

### APOBEC3-interacting sites have undergone diversifying selection in all subtypes/CRFs

**Figure 4.**
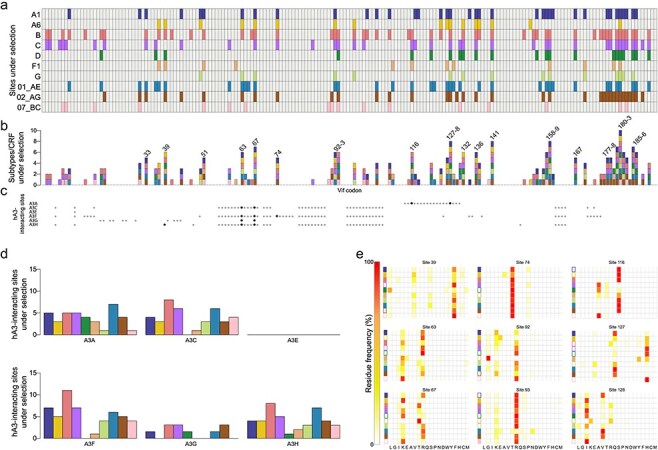
Diversifying selection in *vif* subtypes/CRFs. (a) Cell plot of codons under pervasive diversifying selection in each subtype/CRF and (b) the number of subtypes/CRFs under selection at each *vif* HXB2 codon. Codon numbers are shown when ≥50% of subtypes/CRFs were under selection. (c) hA3-interacting codons corresponding to *vif* HXB2 codons in panel B; black dots indicate codons under selection in ≥50% of subtypes/CRFs. (d) The number of codons under selection at codons interacting with hA3 enzymes across subtypes/CRFs. (e) Frequency distribution of residues at hA3-interacting AA sites under selection in ≥50% of subtypes/CRFs. Filled-in boxes to the left of each graph indicate the subtype/CRF was under selection at the site.

We next ran selection analyses on the alignment and phylogeny for each subtype/CRF. While subtype/CRF consensuses were largely stable over time, many Vif sites have been under diversifying selection, including at hA3-interacting sites. On average, 28.5 codons were under pervasive diversifying selection across subtypes/CRFs, ranging from 12 in F1 to 52 in B (Fig. 4a). The number of sites under selection did not change as a function of the date of the earliest subtype/CRF sample (*P* = 0.075), but did increase slightly with the number of sampled sequences (y = 0.02x, *R*^2^ = 0.71, *P* = 0.001). Sites under selection were distributed across Vif. There was a high density of sites under selection at the 3ʹ end that overlaps the Vpr reading frame, but not at the 5ʹ end overlapping Pol. In total, 84/192 *vif* codons were under selection in at least one subtype/CRF, including 64 (24 hA3-interacting sites) shared between at least two, 25 (7) shared between at least half, and one (codon 181) under selection in all subtypes/CRFs (Fig. 4b). On average, per subtype/CRF there was diversifying selection at 3.8 (min = 1, max = 7) hA3A-interacting sites, 3.8 (0,8) hA3C, 0 hA3E, 5 (0,11) hA3F, 0.9 (1,2) hA3G, and 4.1 (1,8) hA3H (Fig. 4c and d), which correlated with the number of interacting sites in each hA3 enzyme (ρ = 0.89). Given the total number of hA3- and non-hA3-interacting sites, hA3-interacting sites were on average 84.6% (min = 32.9%, max = 153.2%) less likely to be under positive selection than non-hA3-interacting sites in all subtypes/CRFs except A6, for which hA3-interacting sites were 15.6% more likely to be under positive selection. To determine the likely mechanism of selection on each site, we compared the frequency distribution of residues for sequences sampled pre-2000 and post-2000 for each site. Across subtypes/CRFs, 93.3% of sites (including 6/8 hA3-interacting sites) were under balancing selection (Levene’s test, *P* < 0.05; one-sided Kolmogorov–Smirnov test, *P* > 0.05), 7.2% (including 2/8 hA3-interacting sites) were under sweeping selection (Levene’s test, *P* > 0.95; one-sided Kolmogorov–Smirnov test, *P* < 0.05), and only one site was under stabilizing selection (Levene’s test, *P* > 0.95; one-sided Kolmogorov–Smirnov test, *P* > 0.05) (Fig. 4e). To account for potential reversions due to hA3-induced substitutions, for each hA3 site inferred to be under positive selection we derived the consensus residue at the earliest sampling period and calculated the frequency of that residue between 1991–2000 and between 2011–20. We found that for a median of 80% (range = 36.4%–100% across subtypes/CRFs) of sites, the consensus residue was less frequent in the later sampling period. This suggests that the majority of hA3 sites under positive selection were unlikely to be reversions.

### Frequency of subtype/CRF *env* and *gag-pol* hA3-induced substitutions increased over time

**Figure 5.**
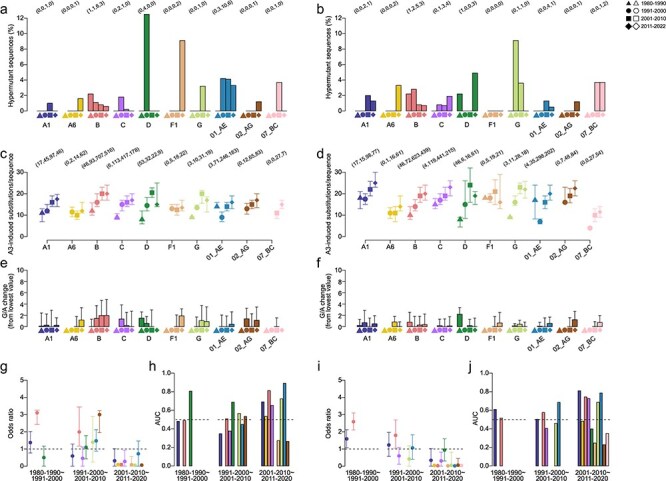
Hypermutation in *env* and *gag-pol* subtype/CRF sequences over time. (a and b) The percentage of hypermutants in each sampling period and subtype/CRF (a) *env* and (b) *gag-pol* sequences. The number of hypermutants are shown parenthetically above. (c and d) The median number of hA3-induced substitutions per sequence for each sampling period and subtype/CRF (c) *env* and (d) *gag-pol* sequences; whiskers indicate 25% and 75% quantiles. The total number of sequences per sampling period and subtype/CRF are shown parenthetically above. (e and f) The relative percentage of A to G nucleotides per sampling period and subtype/CRF (e) *env* and (f) *gag-pol* sequences; whiskers indicate 25% and 75% quantiles. Odds ratio of an hA3-induced substitution at a sampling period being predictive of an hA3-induced substitution at the same site in a later sampling period for (g) *env* and (i) *gag-pol* sequences; an odds ratio of 1 is indicated by the dashed line; whiskers indicate 95% confidence intervals. AUC for predicting the percentage of hA3-induced substitutions in (h) *env* and (j) *gag-pol* sequences at a site in a sampling period as a function of the percentage at a previous sampling period; an AUC of 0.5 is indicated by a dashed line.

We investigated hA3-induced substitutions in subtype/CRF *env* and *gag-pol* alignments over time. We downloaded 3391 *env* sequences and 3277 *gag-pol* sequences with matching accession IDs to Vif sequences in our dataset. This dataset did not include sequences from individuals who had significantly hypermutated Vif sequences (i.e., hypermutants), as these were removed in the curation of Vif sequences. For subtypes/CRFs with ≥ 10 *env* or *gag-pol* sequences in a sampling period, the average percentage of hypermutants ranged, respectively, between 0%–12.5% (Fig. 5a) and 0%–9.1% (Fig. 5b) across sampling periods. The number of hA3-induced substitutions per sequence across subtypes/CRFs increased over time: 8–12 (0.93%–1.40% of codons) between 1980 and 1990, 9–16 (1.05%–1.87%) between 1991 and 2000, 10–21 (1.17%–2.40%) between 2001 and 2010, and 12–20 (1.40%–2.34%) between 2011 and 2022 for *env* sequences (Fig. 5c); and 8–18 (0.56%–1.25%), 4–18 (0.28%–1.22%), 10–24 (0.70%–1.67%), and 12–25 (0.80%–1.74%) for *gag-pol* sequences (Fig. 5d). The average increase was 2.48 for *env* and 2.52 for *gag-pol*. When only RNA-derived sequences were analyzed, there were similar percentages of hypermutants per subtype/CRF (0%–6.2%, Mann-Whitney U test, *P* > 0.11) and the average number of hA3-induced substitutions per sequence was also similar (6–25, *P* > 0.45) (Supplementary Fig. S9).

As hypermutation results in extensive G-to-A mutations, we next estimated the relative percentage of A and G nucleotides [(A-G)/G] in each subtype/CRF over sampling periods, with the assumption that increased frequencies of hA3-induced substitutions would correspond to increasing A at the expense of G. On average, A residues were 49% (min = 46.2%, max = 51.6%) more frequent than G per *env* sequence across sampling periods, but the median change in relative percentage was <1% over time, decreasing as much as 1.39% in CRF02_AG and increasing as much as 1.90% in F1 (Fig. 5e). In *gag-pol*, A residues were 65.6% (62.7%, 70.5%) more frequent than G per sequence, but similarly to *env* the median change over time was <1%, ranging from a decrease of 0.8% in A6 to an increase of 1.2% in F1 (Fig. 5f). This suggests that even though the average number of hA3-induced substitutions per sequence has increased over time in many subtypes/CRFs, these are not extensive enough to have significantly affected the already skewed A/G nucleotide composition.

We next investigated whether the positions of hA3-induced substitutions were consistent over time. We calculated the odds ratio of a hA3-induced substitution within a sampling period being predictive of a hA3-induced substitution at the same site in a later sampling period. Subtype B *env* and both subtype B and A1 *gag-pol* sequences in 1980–90 were predictive of hA3-induced substitutions in 1991–2000 (odds ratio lower 97.5% confidence interval > 1), while subtype B and CRF02_AG *env* and only subtype B *gag-pol* hA3-induced substitution in 1991–2000 were predictive of hA3-induced substitutions in 2001–10 (Fig. 5g and i). hA3-induced substitutions in 2001–10 were not predictive of hA3-induced substitutions in 2011–22 for any subtype/CRF. We used the AUC to predict the percentage of sequences with an hA3-induced substitution at a site in a sampling period as a function of the percentage at a previous sampling period. The AUC was more often significant (AUC > 0.5) but not always consistent with the odds ratios for *env* (Fig. 5h) and *gag-pol* (Fig. 5j). In particular, 6/9 *env* and 5/9 *gag-pol* subtypes/CRFs were significant for 2011–22 as a function of 2001–10. Therefore, while hA3-induced substitutions occasionally persisted at the same position over time, most hA3-induced substitutions were ephemeral.

**Figure 6.**
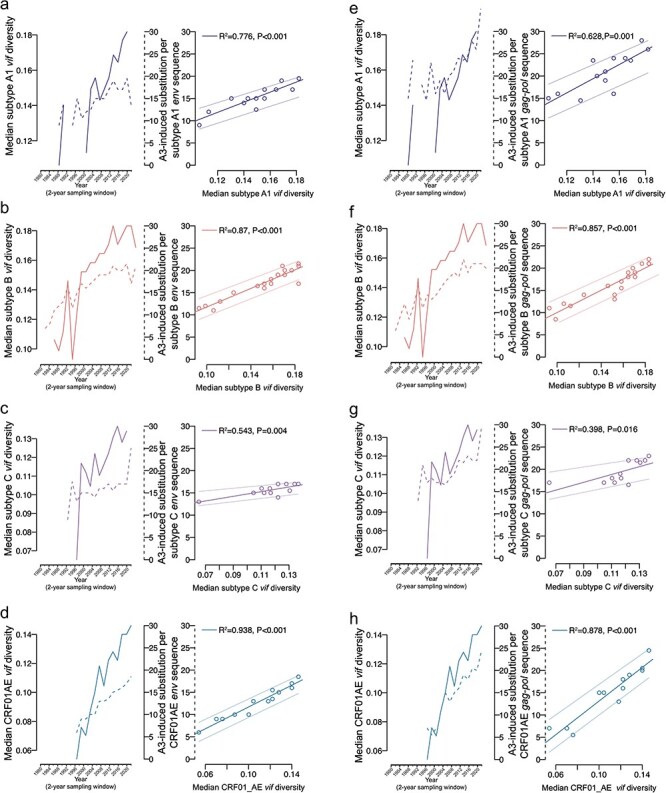
hA3-induced substitutions per sequence as a function of *vif* diversity. The average number of hA3-induced substitutions per *env* sequence and median *vif* pairwise diversity in 2-year sampling time-windows for subtypes (a) A1, (b) B, (c) C, and (d) CRF01_AE. The number of hA3-induced substitutions per *gag-pol* sequence and median *vif* pairwise diversity in 2-year sampling time-windows for subtypes (e) A1, (f) B, (g) C, and (h) CRF01_AE. Best-fit regression lines (darker line) and 95% confidence intervals (lighter lines) are shown, as well as the R^2^ and *P*-value for each best-fit regression.

Lastly, because Vif is in competition with hA3 enzymes, we hypothesized that *vif* diversity would have a negative effect on the number of hA3-induced substitutions occurring over time in *env* and *gag-pol*. We investigated the temporal dynamics between *vif* diversity and hA3-induced substitutions for subtypes A1, B, and C and CRF01_AE by estimating *vif* diversity and hA3-induced substitutions per *env* and *gag-pol* sequence for 2-year time-windows. We restricted analyses to only these subtypes/CRFs due to the limited number of sequences in 2-year time-windows in other subtypes/CRFs. The number of hA3-induced substitutions per *env* (Fig. 6a–d) and *gag-pol* (Fig. 6e–h) sequence increased significantly as a function of median *vif* diversity in each subtype/CRF, although the strength and slope of the relationship was lower for subtype C *env* and *gag-pol*. Therefore, in the best sampled subtypes/CRFs, the frequency of hA3-induced substitutions typically corresponded with *vif* diversity, although with some subtype-specificity. This suggests that diversification in Vif has not adaptively attenuated hA3-mediated substitutions in the period studied.

## Discussion

Our extensive analysis of Vif sequence diversity and its evolutionary response to hA3-mediated effects included 4612 Vif AA and nucleotide sequences, 3391 *env* nucleotide sequences, and 3277 *gag-pol* nucleotide sequences spanning seven subtypes and three CRFs sampled between 1980 and 2022. We found that despite considerable polymorphisms in Vif proteins, as well as extensive diversifying selection, only 2% of Vif consensus sites changed over time. Many sites under diversifying selection had been identified as structurally important to counteracting hA3, including 30 that were under selection in the majority of subtypes/CRFs; and about one-third of consensus sites with mismatches over time were at hA3-interacting sites. However, positive selection and consensus changes were typically less likely to occur at hA3-interacting than non-hA3-interacting sites. Furthermore, diversification at hA3-interacting sites did not curtail hA3 activity on *env* or *gag-pol*, which experienced an increasing number of hA3-induced substitutions per sequence over time. This signifies a prevalence for balancing selection in Vif, wherein sites tolerate diversity without ceding to sweeping selection and therefore consensus changes. To this point, the frequency of consensus residues at sites, including hA3-interacting sites, that were mismatched between sampling periods was on average 20% lower than at sites not mismatched, demonstrating that while diversity at hA3-interacting sites is possible in Vif, there has been modest selective advantage to more effectively combating hA3-mediated immune pressure. Additionally, we found that the number of mismatches across consensus sequences occurring in predicted CTL epitopes was consistent with CTL epitope coverage of the Vif protein, providing no evidence in our dataset of CTL-driven diversification in Vif. Taken together, our population-level analysis of Vif diversification suggests that inefficient counteraction of hA3 editing may have conferred a functional advantage for sporadically increasing HIV-1 genome diversity.

Patterns of diversity over time showed slight differences across subtypes/CRFs. While most saw a steady increase in MRCA divergence, pairwise diversity, and percentage of polymorphic sites, the rate at which this occurred was much higher in, for example, subtype B and CRF01_AE compared to subtype C. In contrast, diversity fluctuated minimally, rather than consistently increased, in subtypes D and G; however, this could have been influenced by small sample sizes. Interestingly, differences in temporal patterns of diversity had no effect on consensus shifts: there were as many as seven sites with mismatches between consensus sequences corresponding to each sampling period in subtype D compared to 0–6 in subtype B and 1–3 in CRF01_AE. The most commonly observed mechanism of selection across subtypes/CRFs and sites was balancing selection, wherein the frequency of the consensus residue was relatively low. The relatively low frequencies of consensus residues revealed widespread diversity at particular sites. This is consistent with recent work on sequence diversity over time in Env, Gag, and Pol proteins (Lewitus et al. 2024) and also highlights that Vif mutants can be nearly as heritable as those carrying a consensus residue.

We found widespread diversifying selection at hA3-interacting sites, but no apparent effect on the rate of hA3-induced substitutions in *env* or *gag-pol*. The number of hA3-induced substitutions per sequence tended to increase over time and was correlated with *vif* pairwise diversity, including at hA3-interacting sites. HIV-1 genomes, and lentiviral genomes generally, are adenine-rich (Rambaut et al. 2004), likely as a result of either targeted G-to-A mutations (Zhang et al. 2003, Harris 2008, Keele et al. 2008, Malim 2009) or the infidelity of reverse transcriptase (Deforche et al. 2007), which can be created by imbalances in dNTP pools (Bebenek et al. 1992). The limited effect of increasing hA3-induced substitutions on A/G ratios suggests that, at the time in lentivirus history when HIV-1 emerged, hA3-guided mutation may no longer have been driving adenine richness. As hA3 deaminases have the capacity to render lentiviral sequences inactive, it is generally assumed that Vif evolved to prevent their incorporation into virions (Sheehy et al. 2003). Consequently, it has been assumed that nonlethal G-to-A mutations are rare (Armitage et al. 2012) and that the frequent occurrence of hypermutation in individuals (Janini et al. 2001, Kieffer et al. 2005) can be explained by CTL-driven selection for Vif variants that interact suboptimally with hA3 (Rousseau et al. 2008, Armitage et al. 2012). Our results do not support this characterization. Instead, we confirm an ample capacity of Vif to diversify at hA3-interacting sites concomitant with increasing proportions of hA3-induced substitutions in viable viruses, as well as limited evidence of excessive mutations in CTL epitopes. Alternatively, *in vivo* evidence of Vif variants that fail to neutralize hA3 activity (Simon et al. 2005) and the prevalence of rapidly diversifying Env sites in hA3 motifs (Wood et al. 2009) suggest that contemporary Vif proteins may not in fact adapt to optimize their counteraction of hA3 editing completely, but to maintain a suboptimal counteraction that may contribute advantageously to HIV-1 genome diversification through sporadic hA3-induced substitutions (Mulder et al. 2008, Fourati et al. 2010, Kim et al. 2010). Accordingly, we showed that *vif* diversification has not attenuated hA3-induced substitutions in *env* or *gag-pol* over time, but rather positively correlated with it. Thus, selection may have favored an inefficiency to counteract hA3-induced substitution that has allowed HIV-1 to exploit hA3-mediated genome diversification.

Restriction factors such as hA3, when allowed to operate successfully, increase viral inactivation, underscoring a possible role for hypermutation in lowering viremia (Pace et al. 2006). Consequently, preventing degradation of hA3 by inhibiting Vif-hA3 interaction (Harris and Liddament 2004, Huthoff et al. 2009, Zuo et al. 2012) and increasing encapsidation of hA3 through overexpression (Peng et al. 2006) have been advanced as potential therapeutic strategies (Jern et al. 2009). However, reduced *in vitro* viral growth of hypermutated compared to non-hypermutated HIV-1 in the presence of the antiviral drug lamivudine suggests that hypermutation may facilitate viral escape (Mulder et al. 2008). Our results show, at the population level, that there has been an increase in hA3-induced substitutions per sequence over time, but any corresponding decrease in viremia has been elusive (Lewitus and Rolland 2022). We find that changes in Vif consensus sequences over time were typically less likely to occur at hA3- than non-hA3-interacting sites, indicative of limited selection pressure for Vif to evade hA3-mediated responses. Furthermore, the positive relationship between *vif* diversity and the rate of hA3-induced substitutions in *env* and *gag-pol* corroborates a potential role for Vif mutants that passively appropriate hA3 to help generate a genetic reservoir for viral escape from antiviral drugs (Mulder et al. 2008). This may be a cause for concern for therapeutic strategies targeting Vif-hA3 interactions.

Our work was limited by the availability of HIV-1 sequences. Disproportionately more sequences deposited post-2000 and historical biases toward sampling (and depositing) subtype B and against subtype C sequences necessarily skewed our dataset. Hypermutated sequences may not typically be submitted to GenBank and the criteria used by researchers to exclude sequences are unknown, which may have biased our dataset toward nonhypermutants. Although previous work has shown that undersampling HIV-1 subtype/CRF sequences can have little effect on inferring diversity estimates (Lewitus et al. 2024), the distribution (and in some cases paucity) of sequences available may have affected our analyses of subtype/CRF Vif diversity and *env* and *gag-pol* hA3-induced substitution. We found small differences in diversity estimates in down-sampled alignments, on average <5% change, and a minor effect of the number of sequences available on the number of sites under selection. We were also not able to ascertain host clinical data on the HLA types of the individuals from whom the sequences were sampled, which could affect hA3 enrichment.

We have shown that the evolution of Vif in the decades since HIV-1 was recognized differs between subtypes/CRFs and over time. There was a nearly 10-fold difference in AA substitution rates between subtypes/CRFs and pairwise diversity increased by an average of 65% between 1980 and 2022. We further found that the number of hA3-induced substitutions in *env* and *gag-pol* increased as a function of *vif* diversity over time. This interplay between *vif* diversification and hA3-induced substitutions in *env* and *gag-pol* reveals a complex relationship that may undermine the putative role of natural restriction factors in HIV-1 evolution. As certain properties of HIV-1 evolution may emerge only at the population level, big data analyses of population-level trends in sequence diversity provide a critical counterpart to intra-individual studies in understanding novelties in outbreaks and the logic of therapeutic interventions.

## Supplementary Material

veae108_Supp

## Data Availability

The data underlying this article are available in the article and in its online supplementary material.
