# Supplementary material for: HIV-1 Vif global diversity and possible APOBEC-mediated response since 1980
Source: Virus Evol. 2024 Dec 12;11(1):veae108. doi: 10.1093/ve/veae108 (PMC11781276; doi:10.1093/ve/veae108)
Supplement: veae108_Supp [file veae108_supp.zip › suppl_data/Vif_SubtypeDiversity_Revision2_accepted_SI_v2.docx]

Supplementary Information: HIV-1 Vif global diversity and possible APOBEC-mediated response since 1980

Eric Lewitus^1,2^${}^{*}$, Yifan Li^1,2^, Morgane Rolland^1,2^

^1^U.S. Military HIV Research Program, Walter Reed Army Institute of Research, Silver Spring, MD

^2^Henry M. Jackson Foundation for the Advancement of Military Medicine, Inc., Bethesda, MD

*Corresponding author: [elewitus@hivresearch.org](mailto:elewitus@hivresearch.org)

### Supplementary Data

**Table S1: Median MRCA divergence and pairwise diversity by sampling period, amino acid substitutions per year, and best-fit growth models for subtype/CRF Vifs.**

**Table S2: Vif amino acid sites and evidence for hA3 interaction.**


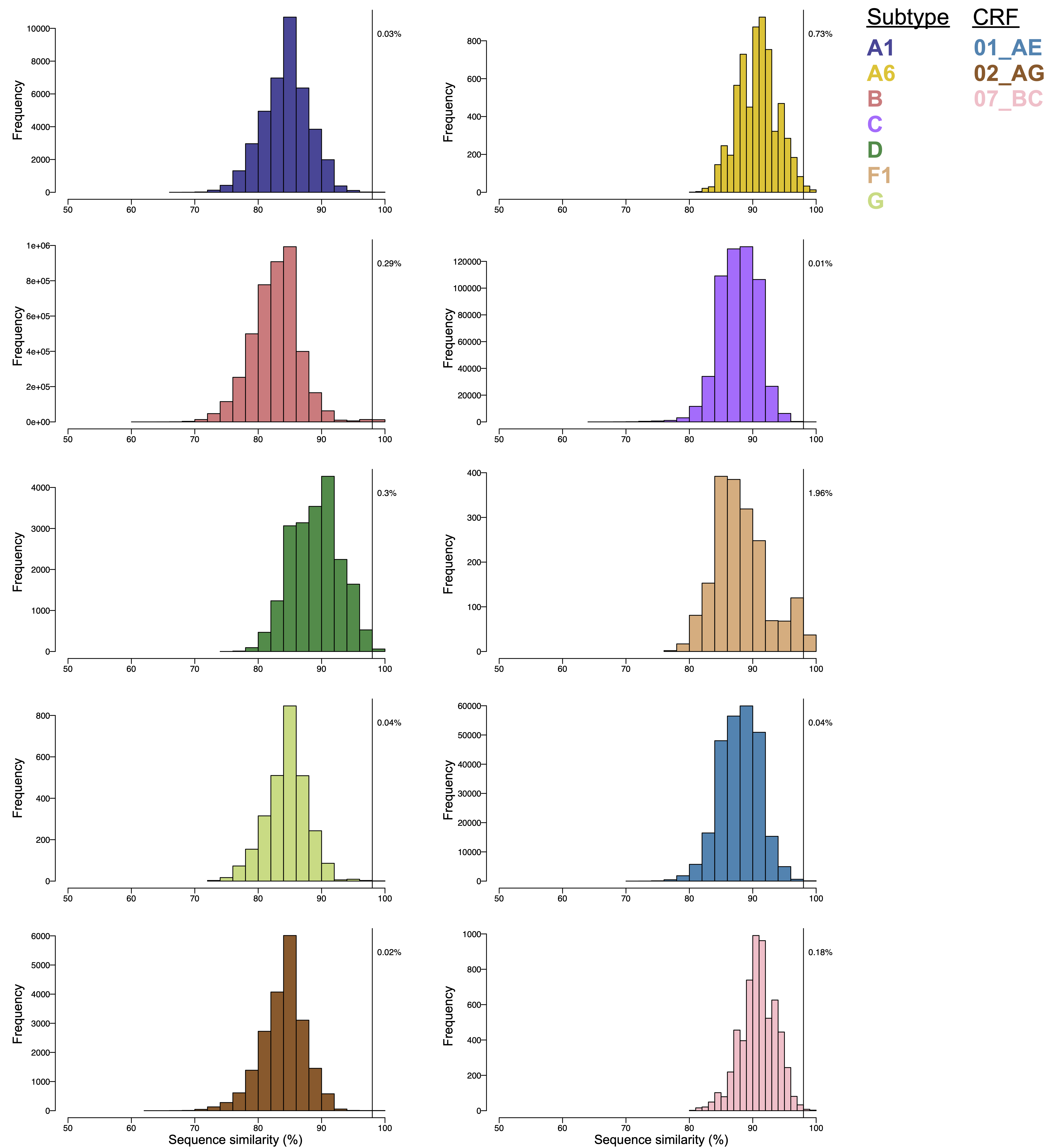


**Figure S1: Subtype/CRF sequence similarity.** Histograms of sequence similarity for each subtype/CRF (see legend). A vertical line is shown at a 98% threshold and the percentage of sequence pairs excluded at that threshold is noted.


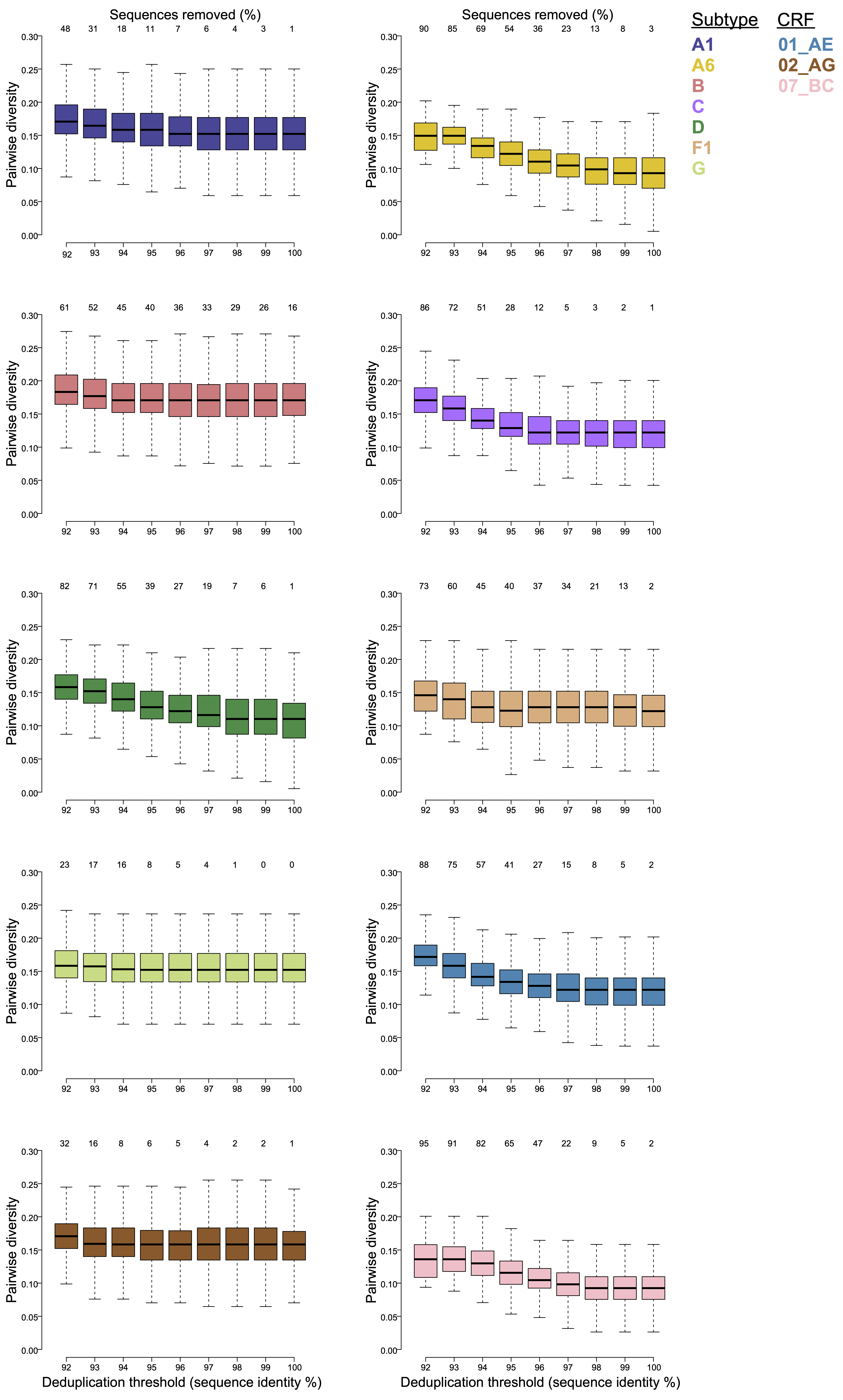


**Figure S2: Subtype/CRF pairwise diversity at sequence identity thresholds.** Boxplots of the pairwise diversity for subtype/CRF sequences included at different sequence similarity thresholds. The percentage of sequences excluded at each threshold are noted above.


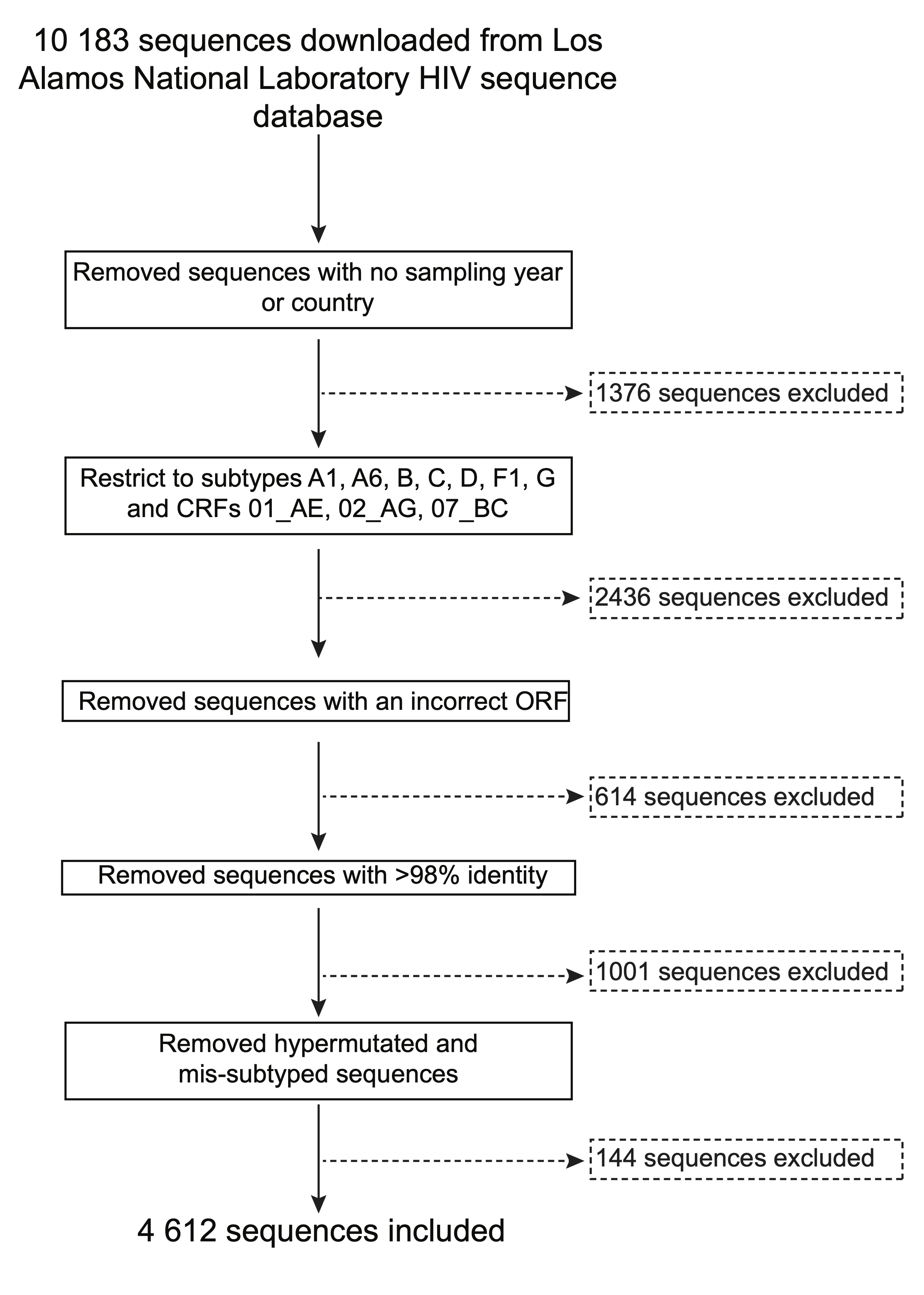


**Figure S3: Workflow for screening Vif amino acid sequences..**


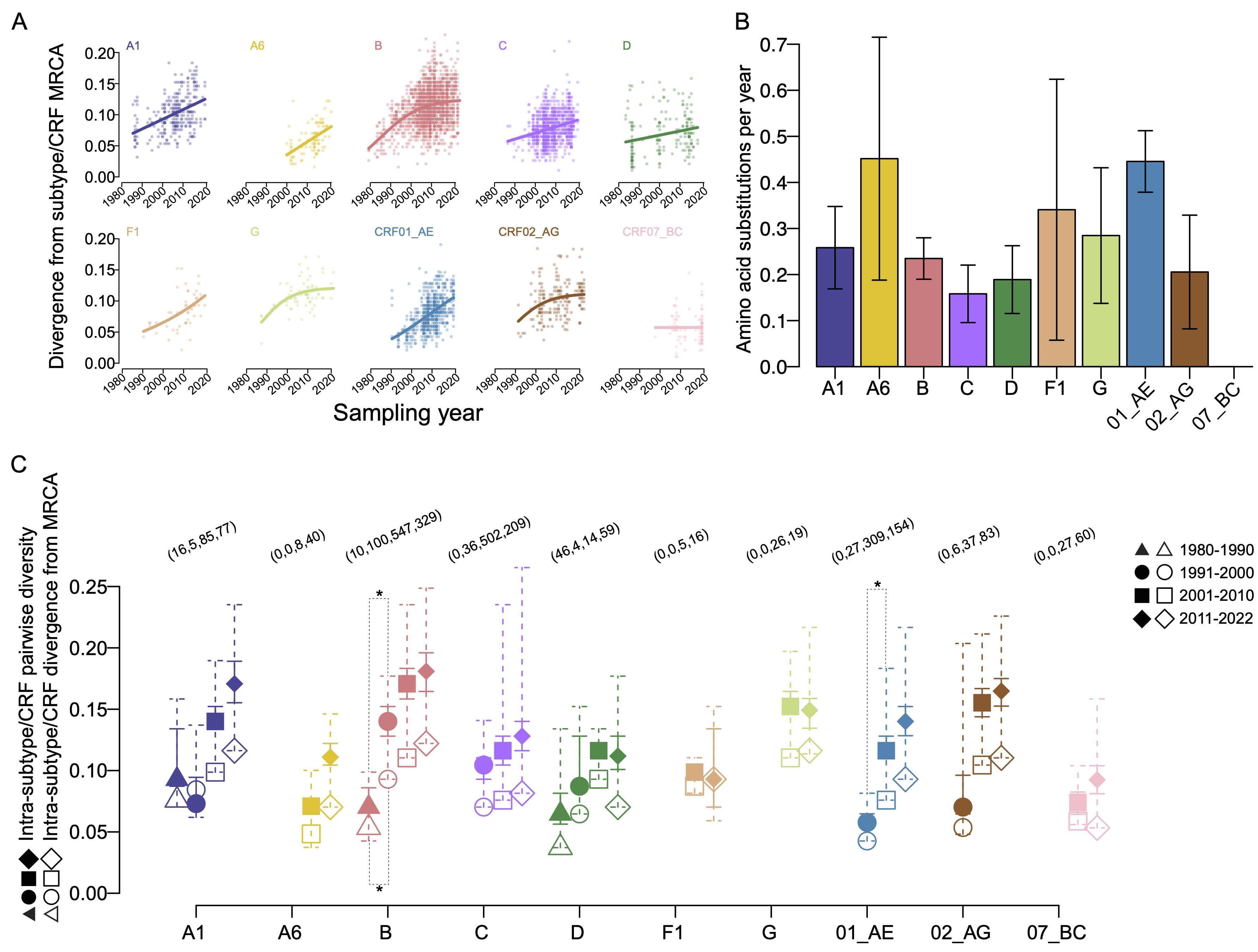


**Figure S4: Vif MRCA divergence and pairwise diversity of RNA-derived sequences.** For RNA-derived sequences in each subtype/CRF, (A) fitted growth curves to MRCA divergence, (B) mean AA substitutions per year (whiskers indicate 95% confidence intervals), and (C) median MRCA divergence (open shapes) and pairwise diversity (closed shapes) by decade. Solid whiskers indicate 25% and 75% quantiles and dashed whiskers indicate minimum and maximum values of pairwise diversity. Asterisks indicate significant pairwise differences between MRCA divergence (below) and pairwise diversity (above). The number of sequences in each sampling period is shown parenthetically above.


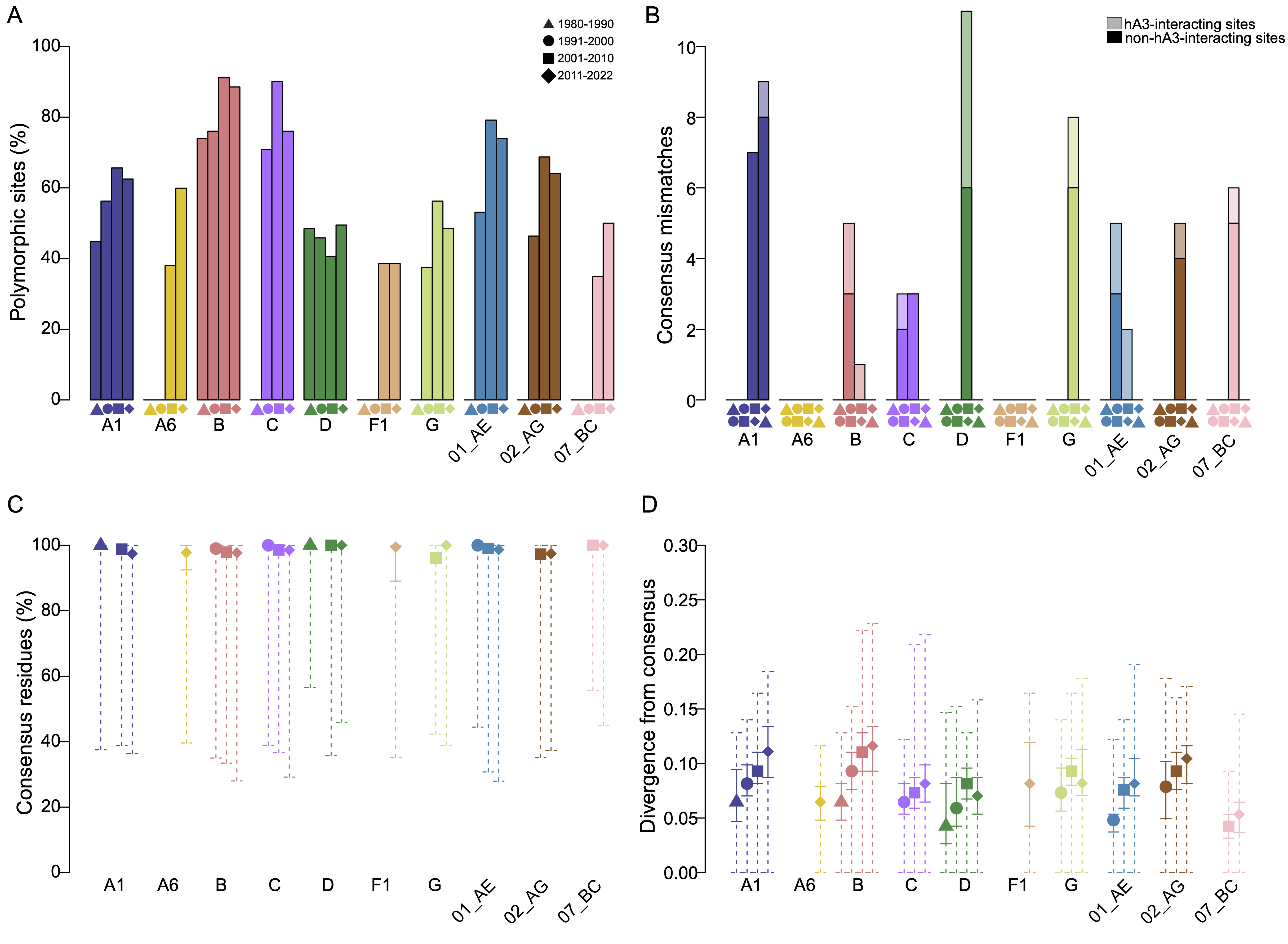


**Figure S5: Vif polymorphisms and consensus changes over time in RNA-derived sequences.** For RNA-derived sequences, (A) The percentage of polymorphic sites in subtype/CRF alignments for 1980-1990 (triangle), 1991-2000 (circle), 2001-2010 (square), and 2011-2022 (diamond). (B) The number of mismatched sites between consensuses for different sampling periods; shapes below each bar indicate the sampling periods being compared. Lighter shades represent mutations at hA3-interacting sites. Sampling periods with fewer than ten sequences are empty in (A) and (B). (C) The median percentage of consensus residues across sites for subtypes/CRFs in each sampling period; solid whiskers indicate 25% and 75% quantiles and dashed whiskers indicate minimum and maximum values. (D) Median divergence from the consensus for each sampling period across subtypes/CRFs; solid whiskers indicate 25% and 75% quantiles and dashed whiskers indicate minimum and maximum values


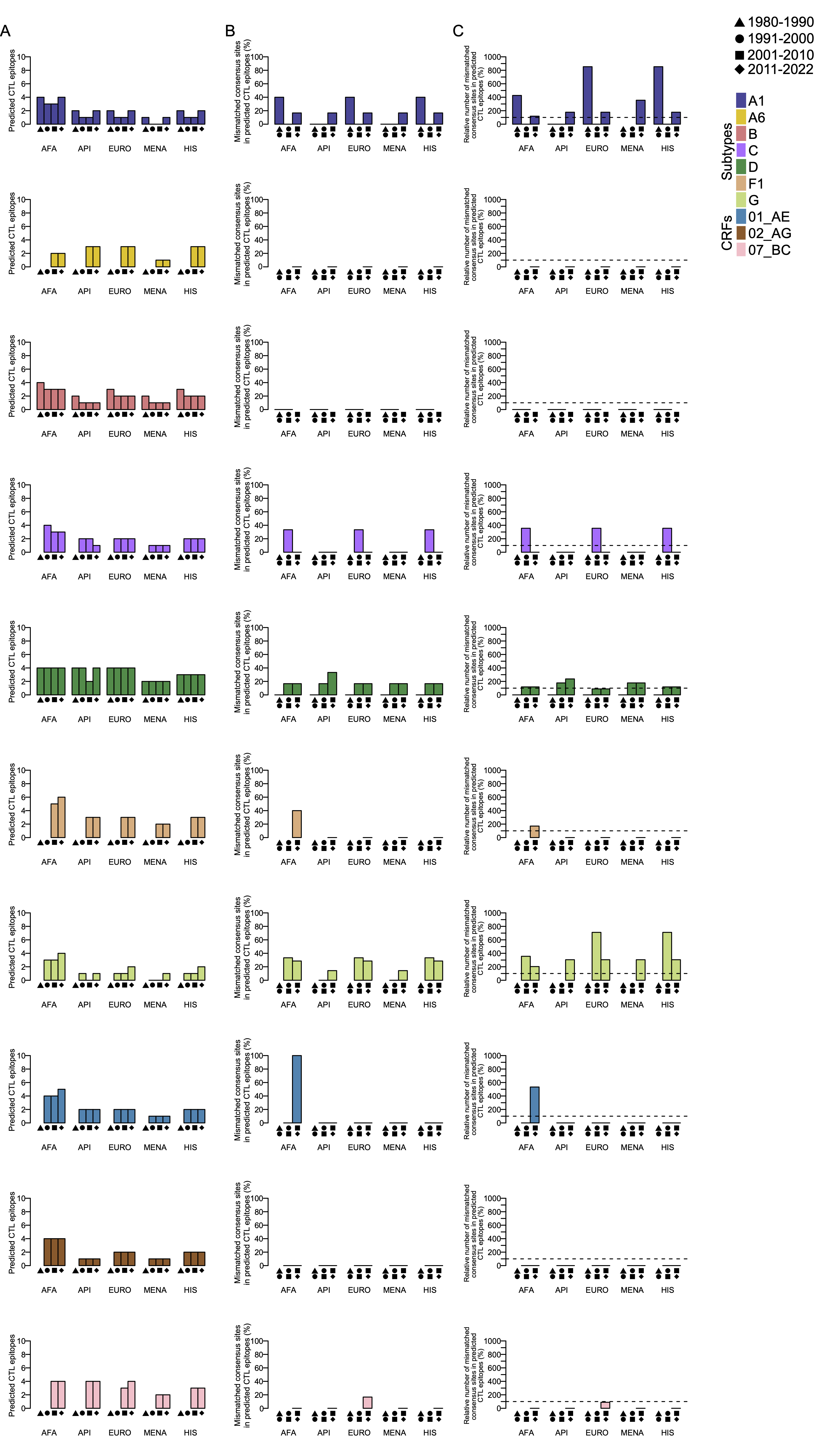


**Figure S6: Predicted CTL epitopes from five major human subpopulations.** (A) The number of predicted CTL epitopes for HLA alleles in each human subpopulation in subtype/CRF consensuses from different sampling periods (see legend). (B) The percentage of the number of mismatched sites between subtype/CRF sampling period (see legend) consensus sequences within predicted CTL epitopes. (C) The relative proportion of panel B compared to the percentage of consensus sequence residues falling within predicted CTL epitopes. Subtype/CRF colors correspond to Figure 1.





**Figure S7: Residue frequencies at sites mismatched across consensus sequences.** Stacked barplot of residue frequencies in ten-year time-windows for sites mismatched across consensus sequences for 1980-1990, 1991-2000, 2001-2010, and 2011-2022. The site is shown above each barplot.


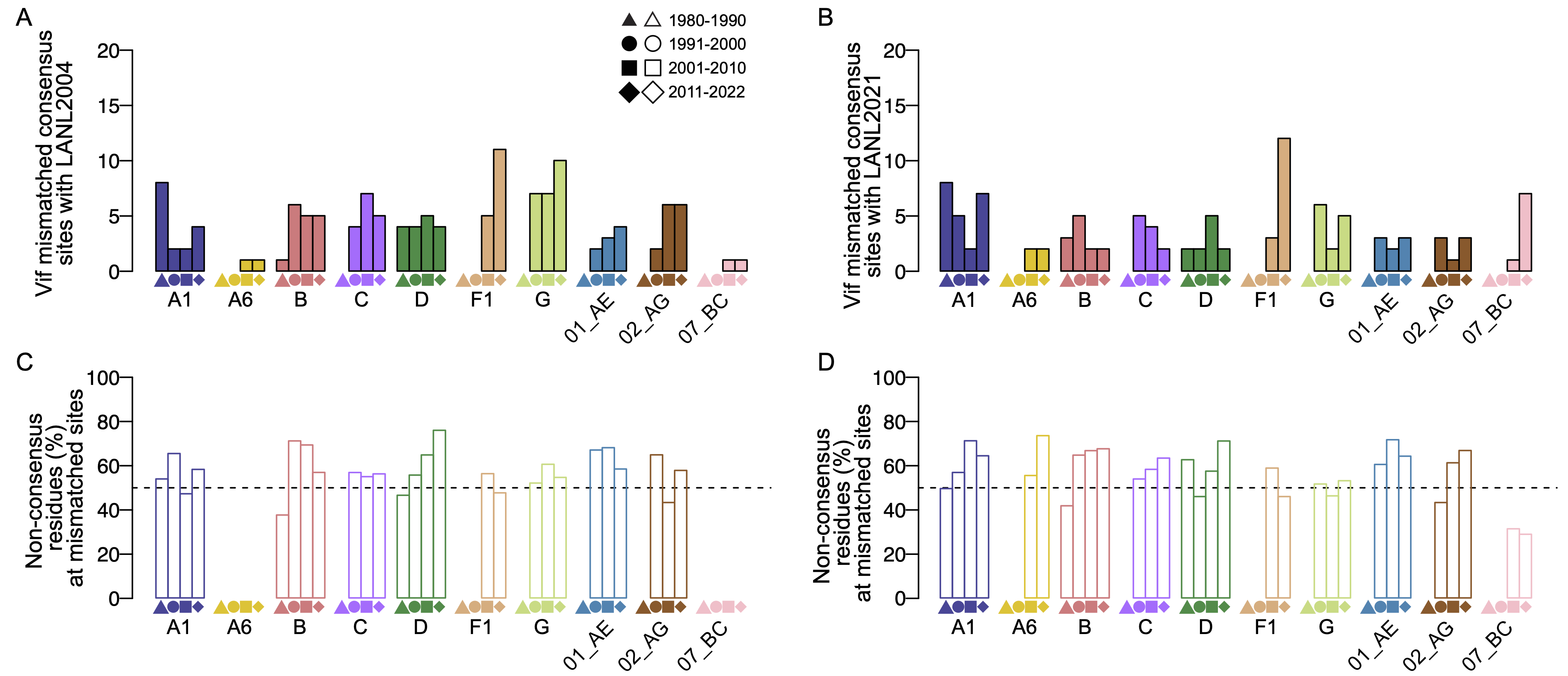


**Figure S8: Consensus mismatches with LANL 2004 and 2021 consensuses.** Mismatched sites between Vif subtype/CRF consensuses for 1980-1990 (triangle), 1991-2000 (circle), 2001-2010 (square), and 2011-2022 (diamond) and the (A) LANL 2004 and (B) LANL 2021 subtype/CRF consensuses. Empty slots indicate sampling periods with fewer than ten sequences available or no LANL consensus available. The median percentage of non-consensus residues in the sampling period alignment at mismatched sites between (C) LANL 2004 and (D) LANL 2021.


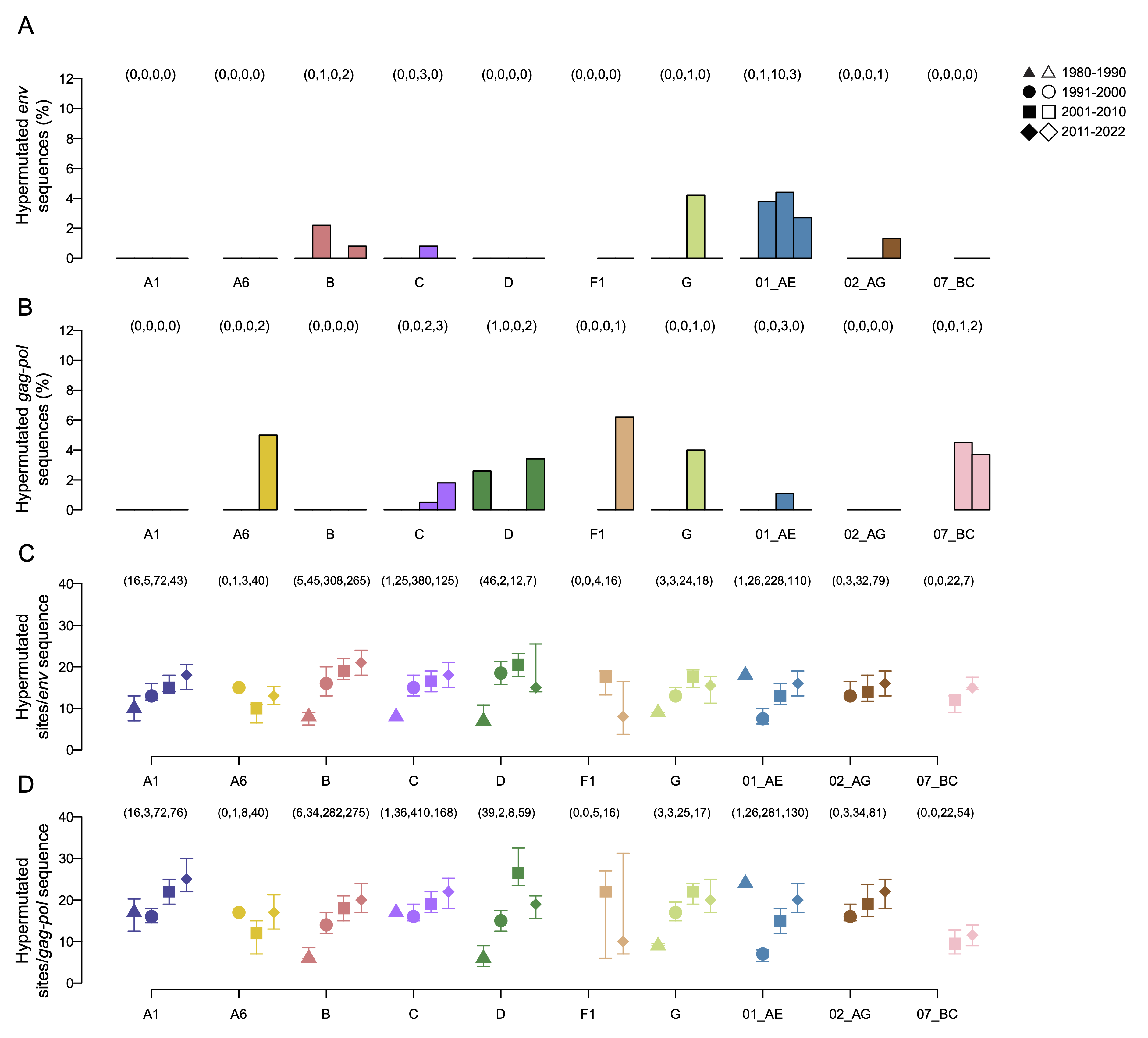


**Figure S9: Hypermutation and hA3-induced substitutions in *env* and *gag-pol* subtype/CRF sequences over time in RNA-derived sequences.** The percentage of hypermutants in each sampling period and subtype/CRF (A) *env* and (B) *gag-pol* sequences derived from RNA. The number of hypermutants are shown parenthetically above. (C,D) The median number of hA3-induced substitutions per sequence for each sampling period and subtype/CRF (C) *env* and (D) *gag-pol* sequences derived from RNA; whiskers indicate 25% and 75% quantiles. The total number of sequences per sampling period and subtype/CRF are shown parenthetically above.
